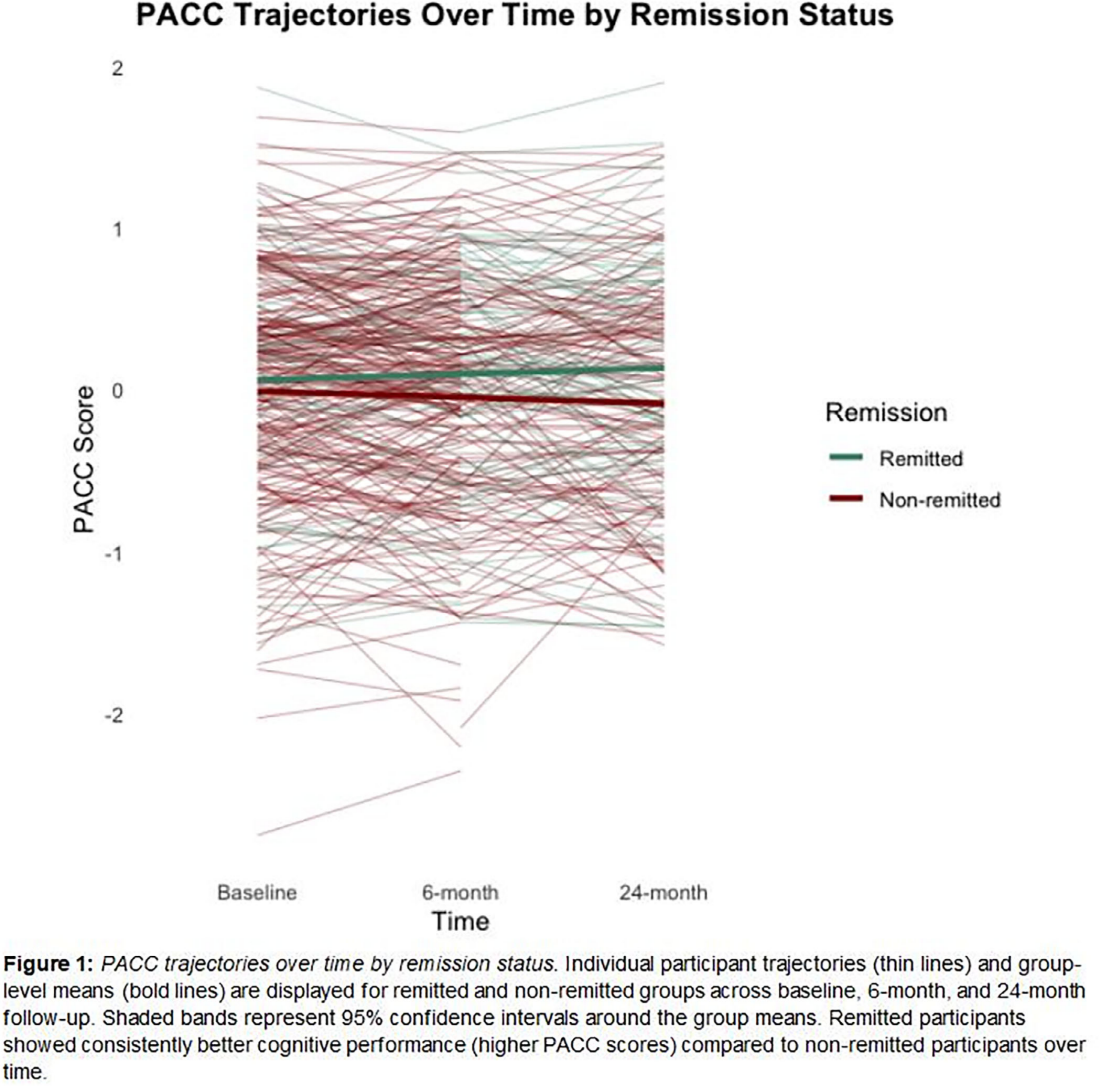# Trajectories of Cognitive Decline over 24 months in Older Adults with Treatment‐Resistant Late‐life Depression at risk for Dementia

**DOI:** 10.1002/alz70861_108863

**Published:** 2025-12-23

**Authors:** Swathi Gujral, Ana Paula Costa, Kayla Conaty, Ashlyn Runk, Elllie Rapp, Keeley M Scullin, Patrick Brown, Helen Lavretsky, Joshua S. Shimony, Benoit H. Mulsant, Aristotle N. Voineskos, Meryl A Butters

**Affiliations:** ^1^ University of Pittsburgh School of Medicine, Pittsburgh, PA USA; ^2^ University of Pittsburgh, Pittsburgh, PA USA; ^3^ University of Pittsburgh Medical Center, Pittsburgh, PA USA; ^4^ Louisiana State University, Baton Rouge, LA USA; ^5^ Columbia University, New York, NY USA; ^6^ UCLA, Los Angeles, CA USA; ^7^ Washington University in Saint Louis School of Medicine, Saint Louis, MO USA; ^8^ Campbell Family Mental Health Research Institute, Centre for Addiction and Mental Health, Toronto, ON Canada; ^9^ University of Toronto, Toronto, ON Canada; ^10^ Toronto Dementia Research Alliance, Toronto, ON Canada; ^11^ Adult Neurodevelopment and Geriatric Psychiatry Division, CAMH, Toronto, ON Canada; ^12^ Department of Psychiatry, Temerty Faculty of Medicine, University of Toronto, Toronto, ON Canada; ^13^ Centre for Addiction and Mental Health, Toronto, ON Canada

## Abstract

**Background:**

Individuals with Late‐life depression (LLD) are at elevated dementia‐risk and those with treatment‐resistant LLD (TRLLD), defined by failing at least two treatment regimens, may be at especially high dementia‐risk. We examined how TRLLD (remission status/severity) predicts cognitive decline over 24‐months.

**Methods:**

Older adults (60+ years) enrolled in OPTIMUM‐NEURO (*N* =375), a multi‐site study of cognitive and brain health decline in those with TRLLD, completed clinical and cognitive assessments at baseline and months 6 and 24. Depression severity was assessed with the Montgomery Asberg Depression Rating Scale (MADRS). Cognition was assessed using the Montreal Cognitive Assessment (MoCA); Repeatable Battery of Neuropsychological Status (RBANS); and subtests from the Delis‐Kaplan Executive Function System (D‐KEFS). Cognitive diagnoses were adjudicated via clinical consensus conferences per 2011 NIA‐AA criteria (Baseline: No cognitive disorder = 178; Mild Cognitive Impairment; MCI = 197; Dementia = 13). Linear mixed‐effects models were used to examine the association between depression severity (including remission) and cognition over time. The primary outcome was the Preclinical Alzheimer’s Cognitive Composite (PACC; Z‐scores: MoCA, RBANS Delayed Recall, RBANS Coding, and D‐KEFS Trail Making Test Condition 4). Models included fixed effects for remission status (MADRS < 10) and time (0, 6, 24 months) on PACC scores, time, and their interaction, controlling for age, sex, education, and race. Random intercepts and slopes for time were modeled for each participant.

**Results:**

At baseline, 53% percent of participants were adjudicated with a research diagnosis of MCI and 17% had remitted depression. Baseline depression severity was moderate among non‐remitted participants (MADRS mean=23, SD=7). Non‐remission and higher depression severity at baseline were associated with worse cognition on average over 24‐months (non‐remission: β= ‐0.108, SE = 0.0534, *p* = 0.044; MADRS: β= ‐0.007, SE = 0.002, *p* = 0.008). Neither baseline depression remission nor severity predicted differential cognitive trajectories over 24 months (all *p* ’s > 0.70).

**Conclusions:**

We have a poor understanding of the role of treatment resistance in increasing dementia risk in LLD. This study showed persistence of depression may predict worse cognitive outcomes in the long‐term but may not specifically accelerate cognitive decline in TRLLD.